# Effects of positive end-expiratory pressure on respiratory function and hemodynamics in patients with acute respiratory failure with and without intra-abdominal hypertension: a pilot study

**DOI:** 10.1186/cc8118

**Published:** 2009-10-05

**Authors:** Joerg Krebs, Paolo Pelosi, Charalambos Tsagogiorgas, Markus Alb, Thomas Luecke

**Affiliations:** 1 Department of Anesthesiology and Critical Care Medicine, University Hospital Mannheim, Faculty of Medicine, University of Heidelberg, Mannheim, Germany, Theodor-Kutzer Ufer, Mannheim, 68165, Germany; 2 Department of Ambient, Health and Safety, University of Insubria, c/o Villa Toeplitz Via G.B. Vico, 46 Varese, 21100, Italy

## Abstract

**Introduction:**

To investigate the effects of positive end-expiratory pressure (PEEP) on respiratory function and hemodynamics in patients with acute lung injury (ALI) or acute respiratory distress syndrome (ARDS) with normal intra-abdominal pressure (IAP < 12 mmHg) and with intra-abdominal hypertension (IAH, defined as IAP ≥ 12 mmHg) during lung protective ventilation and a decremental PEEP, a prospective, observational clinical pilot study was performed.

**Methods:**

Twenty patients with ALI/ARDS with normal IAP or IAH treated in the surgical intensive care unit in a university hospital were studied. The mean IAP in patients with IAH and normal IAP was 16 ± 3 mmHg and 8 ± 3 mmHg, respectively (*P *< 0.001). At different PEEP levels (5, 10, 15, 20 cmH_2_O) we measured respiratory mechanics, partitioned into its lung and chest wall components, alveolar recruitment, gas-exchange, hemodynamics, extravascular lung water index (EVLWI) and intrathoracic blood volume index (ITBVI).

**Results:**

We found that ALI/ARDS patients with IAH, as compared to those with normal IAP, were characterized by: a) no differences in gas-exchange, respiratory mechanics, partitioned into its lung and chest wall components, as well as hemodynamics and EVLWI/ITBVI; b) decreased elastance of the respiratory system and the lung, but no differences in alveolar recruitment and oxygenation or hemodynamics, when PEEP was increased at 10 and 15cmH_2_O; c) at higher levels of PEEP, EVLWI was lower in ALI/ARDS patients with IAH as compared with those with normal IAP.

**Conclusions:**

IAH, within the limits of IAP measured in the present study, does not affect interpretation of respiratory mechanics, alveolar recruitment and hemodynamics.

## Introduction

Over the past decade there has been a marked increase in interest in the role of intra-abdominal pressure (IAP) in critically ill patients [1]. The World Society of Abdominal Compartment Syndrome (WSACS) defined intra-abdominal hypertension (IAH) as a sustained or repeated pathological elevation in IAP of 12 mmHg or more [2,3] observed in 54.4% and 65.0% of medical and surgical critically ill patients, respectively [4]. IAH has been shown to negatively affect various organ functions [1], including the respiratory system [5] and hemodynamics [6].

Acute lung injury (ALI) and adult respiratory distress syndrome (ARDS) are characterized by an increase in elastance of the respiratory system (Estat, RS), mainly attributed to the elastance of the lung (Estat, L). However, alterations in the chest wall elastance (Estat, CW) have also been increasingly described in patients with ALI/ARDS, associated with increased IAP [7]. The distending force of the lung is the transpulmonary pressure (Ptranspul = alveolar pressure minus pleural pressure), which depends on the pressure applied to the airways and Estat, L/Estat, CW. Therefore, if Estat, CW is higher, the same applied airway pressure may result in substantially higher pleural pressure, with lower Ptranspul and less lung distention [1]. Ptranspul, rather than airway pressure, has been shown to be associated with lung stress during mechanical ventilation [8]. Furthermore, positive end-expiratory pressure (PEEP) has been reported to improve respiratory system, lung and chest wall mechanics, alveolar recruitment, and gas exchange in ALI/ARDS patients with increased IAP compared with patients with normal IAP [7]. More recently, in a study including mainly extrapulmonary ALI/ARDS patients, a strategy titrating PEEP according to end-expiratory Ptranspul and not to the absolute PEEP value has been shown to improve oxygenation, respiratory system mechanics and revealed a trend towards better survival [9]. On the other hand, an increase in pleural pressure, due to increased Estat, CW and IAP may negatively influence hemodynamics [1,10]. In fact, the increase in airway pressures by PEEP may be associated with a higher increase in pleural pressure in ALI/ARDS patients with higher IAP resulting in reduced intrathoracic blood volume [11-13].

Increased IAP has been associated with increased extravascular lung water and edema, at least in animal models [14]. However, data are scarce on whether the presence or absence of IAH *per se *may significantly affect respiratory function, esophageal pressure (Pes) and hemodynamics, including extravascular lung water and intrathoracic blood volume. Furthermore, no previous data have been published looking at the effects of PEEP in ALI/ARDS patients with and without IAH. We hypothesized that patients with IAH, as currently defined by the WSACS, were characterized by different respiratory function, extravascular lung water and intrathoracic blood volume, and that PEEP may differently affect the respiratory and hemodynamics response according to IAP level.

Therefore, the present prospective observational pilot study was designed to assess the consequences of either normal IAP (< 12 mmHg) or IAH (defined as IAP ≥ 12 mmHg) on the effects of PEEP on gas exchange, respiratory mechanics and hemodynamics in 20 patients with ALI/ARDS.

## Materials and methods

### Patients

Following approval of the local ethics committee, written informed consent was obtained from each patient's next of kin. Every mechanically ventilated patient with ALI or ARDS meeting American-European Consensus Conference (AECC) criteria [15] was considered eligible for the study. IAP was measured according to WSACS recommendations [2] (see below) and patients were prospectively stratified into two groups: normal IAP (< 12 mmHg) and IAH (IAP ≥ 12 mmHg, IAH) groups on at least three consecutive measurements within a 12-hour time interval. Exclusion criteria were the following: age younger than 18 years, mechanical ventilation for more than five days, pregnancy, severe head injury, inherited cardiac malformations, presence of arrhythmias, immunosuppression, end-stage chronic organ failure and expected survival of less than 24 hours. Before interventions were started patients had to be hemodynamically stable (described below). Adequate sedation (Richmond Agitation-Sedation Scale score -5) [16] was ensured with intravenous midazolam (5 to 15 mg/h) and fentanyl (0.5 to 2.5 mg/h) throughout the study. Paralyzing agents were not used. The ventilator was set by the attending physician in the volume-control mode with tidal volumes of 6 ml/kg ideal body weight, an inspiration:expiration ratio of 1:1 and respiratory rate set to keep arterial pH above 7.20. PEEP was set using the ARDS clinical network (ARDSNet) PEEP/fraction of inspired oxygen (FiO_2_) table [17]. Norepinephrine was used if mean arterial pressure (MAP) was below 65 mmHg despite adequate intravascular volume status as defined below. All patients had a triple-lumen central venous catheter (via the subclavian or internal jugular vein) and a thermodilution catheter (5F Pulsiocath™, Pulsion Medical Systems, Munich, Germany) in a femoral artery inserted. The Pulse Contour Cardiac Output monitor (PiCCOplus™) was used for hemodynamic measurements and intravascular volume optimization in all patients as standard of care.

### Study design

In all patients while in the supine position, we measured the IAP, the elastic properties of lung and chest wall (in triplicate) as well as hemodynamics and gas exchange at baseline ventilatory settings and at four different PEEP levels applied as a decremental trial (20, 15, 10, and 5 cmH_2_O). The other ventilator settings were kept constant throughout the entire protocol. Measurements were taken in 30-minute intervals to allow for equilibration of gas exchange and mechanics. At baseline and each level of PEEP, a recruitment maneuver was performed to standardize the history of lung volume [18], in which airway pressure was increased to 40 cmH_2_O for 30 seconds. As the last measurement taken at each level, exhaled volume to zero end-expiratory pressure (ZEEP) was measured. Exhaled volume below PEEP was calculated as exhaled volume to ZEEP minus tidal volume. Another recruitment maneuver was performed immediately following this measurement to reverse potential lung collapse. Termination criteria were as follows: a) reduction in cardiac index (CI) more than 20% or below 2.5 l/min/m^2 ^compared with baseline after PEEP application; b) increase in end inspiratory Ptranspul higher than 27 cmH_2_O [8].

### Intra-abdominal pressure measurements

IAP was measured strictly according to WSACS recommendations [2]. IAP was measured at end-expiration with the patient in the supine position and the transducer zeroed at the level of the mid-axillary line using an instillation volume of 25 ml normal saline in the bladder. Measurements were taken 45 seconds after instillation. The baseline measurements used to stratify patients into normal IAP or IAH group were taken at a level of PEEP set according to the ARDSNet PEEP/FiO_2 _table [17]. No patient changed their initial group taken into account the PEEP value at baseline.

### Respiratory mechanics

Respiratory mechanics were assessed during end inspiratory and end expiratory occlusion as described previously [19]. Tracheal pressure (Ptrach) was obtained using a dedicated catheter (Tracheal Catheter P/N 10635™, Cardinal Healthcare, Dublin, OH, USA) advanced through the endotracheal tube using a sealed sideport connector. For Pes measurement an esophageal balloon catheter (Esophageal Catheter, Cardinal Healthcare, Dublin, OH, USA) was used. Ptrach, Pes and gas flow were measured and recorded by a computerized system incorporated into the mechanical ventilator (AVEA™, Cardinal Healthcare, Dublin, OH, USA). The esophageal balloon was automatically inflated with 0.5 to 1 ml of air. The balloon catheter was first passed by nose into the stomach with its tip 60 cm from the nares and then withdrawn to about 40 cm to measure Pes. Proper balloon position was confirmed in all patients by observing an appropriate change in the pressure tracing as the balloon was withdrawn into the thorax (changes in pressure waveform, mean pressure and cardiac oscillation), as well as by observing a transient increase in pressure during a gentle compression of the abdomen as described previously [9,20]. Volume was obtained by digital integration of the flow signal.

### Static elastance of the total respiratory system, lung, and chest wall

Ptrach and Pes were recorded during a 3 to 4 second airway occlusion at end expiration and end inspiration. Estat, RS was computed as DPtrach/V_T_, where DPtrach is the difference between end-inspiratory and end-expiratory tracheal pressure and V_T _is the tidal volume. Estat, CW was computed as DPes/V_T_, where DPes is the difference between end-inspiratory and end-expiratory esophageal pressure. Estat, L was calculated as Estat, RS - Estat, CW.

### Estimated lung recruitment

The gas volume of collapsed lung units thought to be recruited with PEEP (estimated lung recruitment) was calculated as previously described [21] as the difference in lung volume between PEEP 10, 15 and 20 mmHg, respectively, and PEEP 5 mmHg for the same static respiratory system pressure (20 cmH_2_O) from the static tidal volume-pressure curves obtained at the different PEEP levels. A static respiratory system pressure of 20 cmH_2_O was chosen because this pressure was available for all levels of PEEP. The basic assumption for this estimate of alveolar recruitment relies on the finding that the specific elastance of pulmonary units is near to normal [22], indicating that the elastance decrease with PEEP is likely to be due to the recruitment of new pulmonary units [7].

### Hemodynamic measurements

MAP, stroke volume index, CI, intrathoracic blood volume index (ITBVI) and extravascular lung volume index (EVLWI) were obtained using the Contour Cardiac Output (PiCCOplus™) system (see above). The PiCCO apparatus was calibrated with the intermittent transpulmonary thermodilution technique using three times 20 ml iced saline immediately before the first set of measurements. CI was calculated by the PiCCO monitor from the area under the arterial pulse curve of each heartbeat and from an estimation of systemic vascular resistance based on MAP and a manually entered central venous pressure. Hemodynamic stability was defined as a MAP of more than 65 mmHg, heart rate of less than 130 beats/min and a CI more than 2.5 l/min/m^2^. Intravascular volume status was titrated using the ITBVI aiming at low normal values (800 to 1000 ml/m^2^).

### Statistics

All data are presented as mean ± standard deviation. To test normal distribution, the Kologomorow-Smirnov test was used. To analyse statistical differences at baseline, paired sample t-test was applied. To investigate the effects of PEEP in the two groups, two-way recruitment maneuver analysis of variance was performed. SAS version 9.1.3 (SAS institute, Cary, NC, USA) was used for statistical analysis. On the basis of previous published studies we calculated a sample size of 10 patients per group in order to detect significant differences (power 80%, alpha error 0.05 and beta error 0.2) in gas exchange, respiratory mechanics, and hemodynamics.

## Results

From July 2007 to September 2008, 20 patients meeting AECC criteria for ALI or ARDS with and without IAH were included in the study. As the inclusion rate during the study period was similar we evaluated 10 patients in each group. No patient met the termination criteria during the PEEP trial. The mean IAP in patients with increased or normal IAP was 16 ± 3 mmHg and 8 ± 3 mmHg, respectively (*P *< 0.001). Table 1 summarizes anthropometric characteristics, Simplified Acute Physiology Score (SAPS) II, duration of mechanical ventilation prior to inclusion and outcome for patients included in normal and increased IAP groups. All patients were studied within one week from ALI/ARDS onset. We found no differences in any variable between the two groups except for an increased intensive care unit mortality and the prevalence of extrapulmonary ALI/ARDS in the IAH group. In fact, in the normal IAP group, five out of ten patients had pneumonia, thus classified as primary ALI/ARDS, while the remaining five patients had small bowel perforation (two patients), anastomotic leakage (one patient) and hemorragic shock (two patients) as underlying pathology. Contrasting, in the IAH group all patients were classified as secondary ALI/ARDS, due to peritonitis (two patients), pancreatitis (two patients), anastomotic leakage (one patient), small bowel perforation (one patient), pseudomembranous colitis (one patient), ruptured abdominal aortic aneurysm (one patient), gangrenous cholecystitis (one patient), and chest trauma (one patient).

**Table 1:** Patient characteristics at baseline

	Normal IAP (n = 10)	IAH (n = 10)	*P *value
Sex M/F	7/3	7/3	NS
Age, year	60 ± 9	66 ± 10	NS
BMI	29 ± 4	27 ± 3	NS
Height, cm	168 ± 10	171 ± 9	NS
Weight, kg	83 ± 8	79 ± 8	NS
SAPS II	42 ± 13	48 ± 24	NS
ICU mortality, (%)	20	40	< 0.05
MV before study, days	2.4 ± 2.6	2.7 ± 2.4	NS
ICU stay, days	24 ± 24	12 ± 7	NS
ALI/ARDS_P _vs ALI/ARDS_S_	5/5	0/10	< 0.001

ALI/ARDS_p _= primary ALI/ARDS; ALI/ARDS_s _= secondary ALI/ARDS; BMI = body mass index; F = female; IAH = intra-abdominal hypertension; IAP = intra-abdominal pressure; ICU = intensive care unit; M = male; MV = mechanical ventilation; NS = not significant; SAPS = simplified acute physiology score.

Data are presented as mean ± standard deviation.

### Baseline measurements

**Figure 1:**
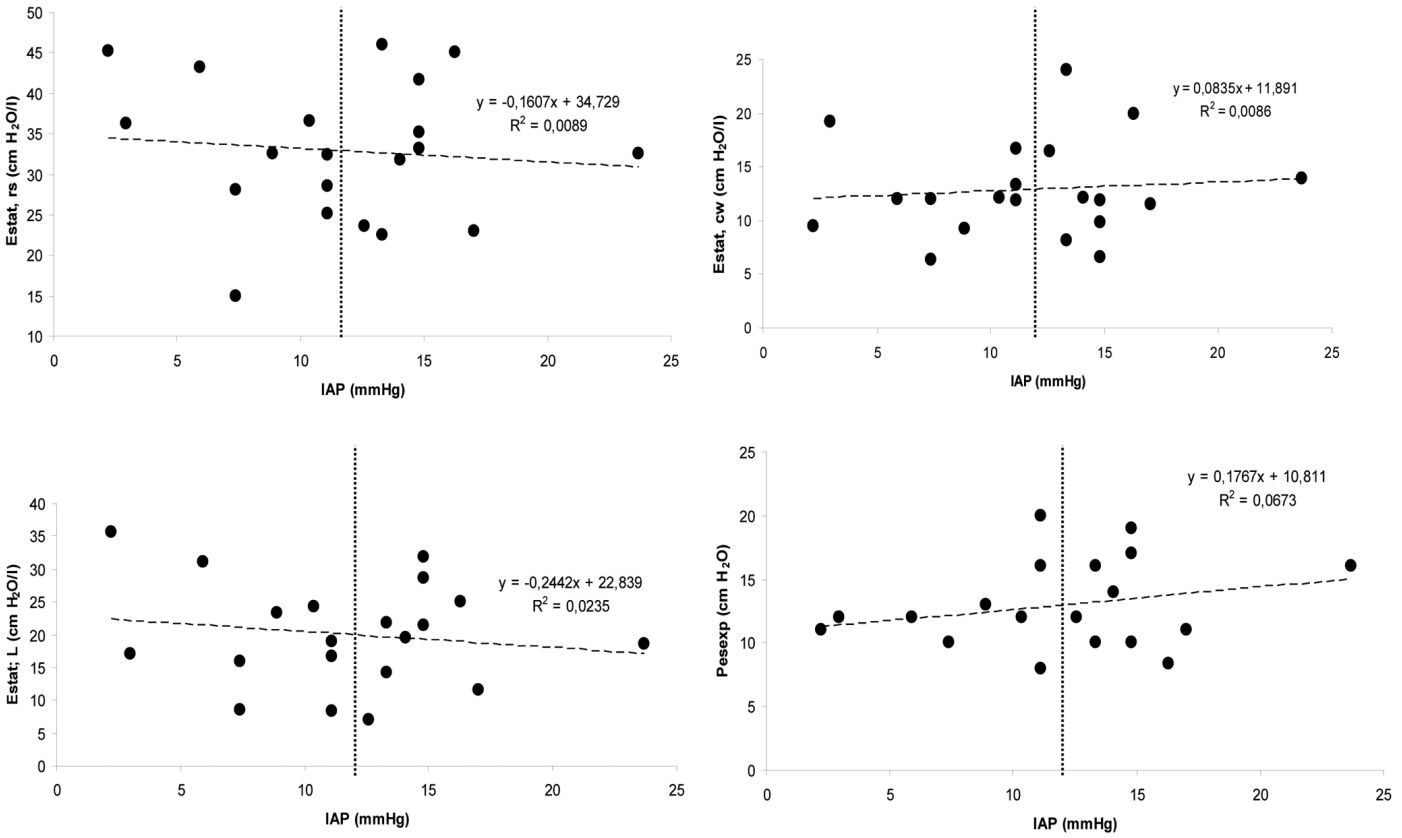
Correlation between intra-abominal pressure (IAP) and static elastance of the respiratory system (Estat, rs; *upper left panel*), static elastance of the chest wall (Estat, CW; *upper right panel*), static elastance of the lung components (Estat, L; *lower left panel*), and transpulmonary end-expiratory pressure (Pes exp) (*lower right panel*) at baseline. The vertical dashed lines at 12 mmHg separate the groups with normal IAP (< 12 mmHg) and intra-abdominal hypertension (> 12 mmHg).

At baseline no significant differences were found in ventilator settings, gas exchange, respiratory mechanics, partitioned into its lung and chest wall components, as well as hemodynamics and EVLWI/ITBVI between normal IAP and IAH groups (Table 2). Ptranspul at end inspiration (Ptranspulinsp) and end expiration (Ptranspulexp) were similar in the IAH and the normal IAP groups (inspiration: 8 ± 4 cmH_2_O versus 9 ± 5 cmH_2_O, *P *= 0.79; expiration: -1 ± 3 cmH_2_O versus 0 ± 3 cmH_2_O, *P *= 0.76, respectively). No significant correlation was found between IAP and Estat, RS, or its lung and chest wall components (Figures 1a to 1c). Furthermore, there was no correlation between IAP and the Pes at end-expiration (Figure 1d).

**Table 2:** Ventilatory setting, gas exchange, respiratory mechanics and hemodynamic variables at baseline

	Normal IAP (n = 10)	IAH (n = 10)	*P *value
**Ventilatory setting**			
V_T _(ml/kg IBW)	5.3 ± 0.4	5.6 ± 0.3	0.178
RR, breaths/min	18 ± 3	16 ± 3	0.129
PEEP, cmH_2_O	9.1 ± 1.7	9.6 ± 1.8	0.532
**Gas exchange**			
PaO_2_/FiO_2_	203 ± 51	180 ± 77	0.479
PaCO_2_, mmHg	44 ± 9	44 ± 9	0.875
PH	7.35 ± 0.09	7.39 ± 0.05	0.234
**Mechanics**			
Pplat, cmH_2_O	23 ± 4	24 ± 4	0.623
Pes insp, cmH_2_O	18 ± 4	19 ± 3	0.378
Pes exp, cmH_2_O	12 ± 3	13 ± 4	0.554
Ptranspul insp, cmH_2_O	9 ± 5	8 ± 4	0.796
Ptranspul exp, cmH_2_O	0 ± 3	-1 ± 3	0.760
Estat, RS, cmH_2_O/l	32.3 ± 8.8	33.4 ± 8.7	0.774
Estat, L, cmH_2_O/l	20 ± 8.8	20 ± 7.6	0.988
Estat, CW, cmH_2_O/l	12.3 ± 3.7	13.4 ± 5.4	0.567
VexhaledZEEP, ml	236 ± 139	185 ± 98	0.402
**Hemodynamics**			
HR, beats/min	89 ± 26	82 ± 24	0.545
MAP, mmHg	80 ± 13	76 ± 7	0.463
CI, l/min/m^2^	4.5 ± 1.6	3.4 ± 1.1	0.097
ITBVI, ml/m^2^	991 ± 334	1060 ± 163	0.562
EVLWI, ml/m^2^	10.7 ± 5.7	6.7 ± 1.9	0.059

Estat, CW = static chest wall elastance; Estat, L = static lung elastance; Estat, RS = static respiratory system elastance; EVLWI = extravascular lung water index; FiO2 = fraction of inspired oxygen; HR = heart rate; IAH = intra-abdominal hypertension; IAP = intra-abdominal pressure; IBW = ideal body weight; ITBVI = intrathoracic blood volume index; MAP = mean arterial pressure; PaCO2 = partial pressure of arterial carbon dioxide; PEEP = positive end-expiratory pressure; Pes insp = inspiratory esophageal pressure; Pes exp = expiratory esophageal pressure; Pplat = inspiratory plateau pressure; Ptranspulexp = expiratory transpulmonary pressure; Ptranspul insp = inspiratory transpulmonary pressure; RR = respiratory rate; Vexhaled ZEEP = exhaled volume from PEEP to zero end-expiratory pressure; V_T _= tidal volume.

Data are presented as mean ± standard deviation.

### Effects of PEEP

**Figure 2:**
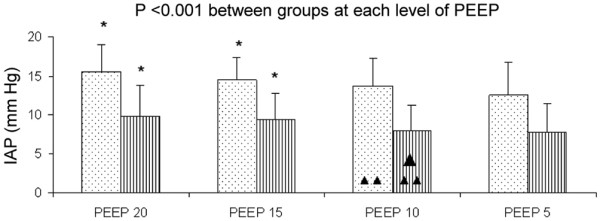
Effect of PEEP on IAP in patients with normal IAP (shaded bars) and intra-abdominal hypertension (dotted bars).  Asterisk: *P *< 0.05 vs positive end-expiratory pressure (PEEP) 5; Single Triangle: *P *< 0.05 PEEP 10 vs PEEP 15; Double Triangle: *P *< 0.05 PEEP 10 vs PEEP 20. Data are presented as mean ± standard deviation. IAP = intra-abdominal pressure.

**Figure 3:**
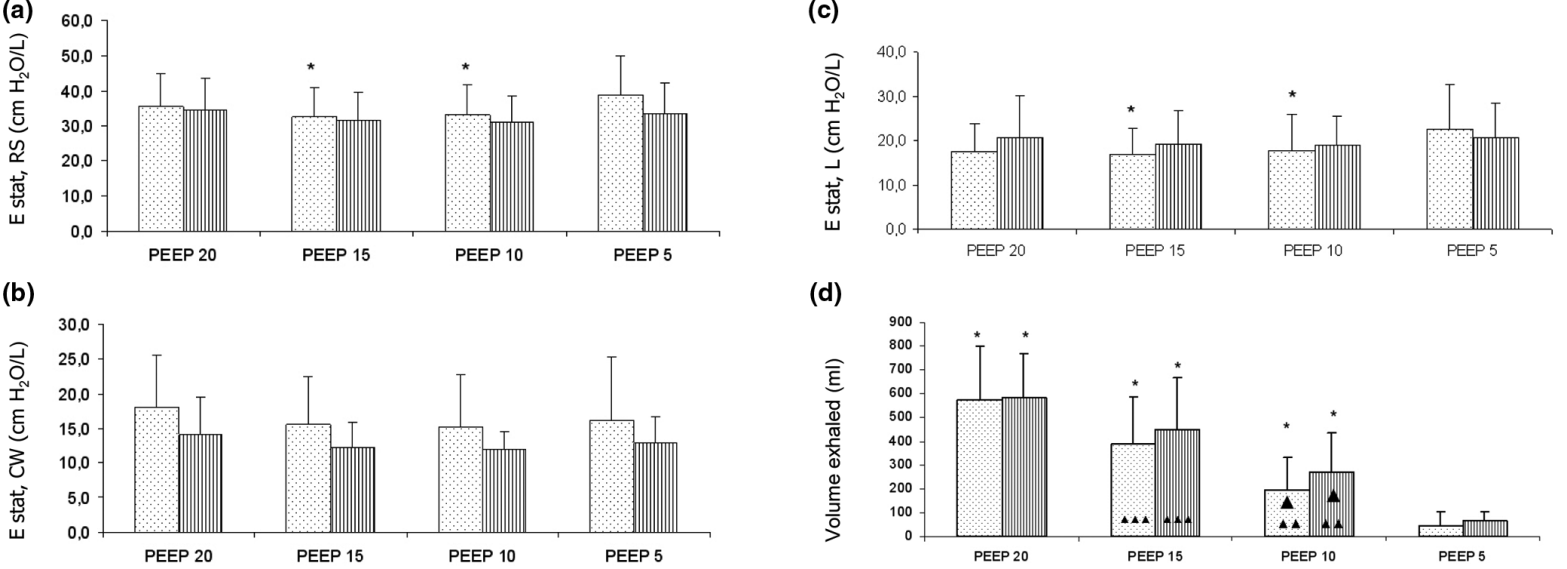
Effect of PEEP on the (a) static elastance of the respiratory system (Estat, rs), (b) static elastance of the chest wall (Estat, CW), (c) static elastance of the lung components (Estat, L) and (d) the volume exhaled from PEEP to ZEEP in patients with normal IAP (shaded bars) and intra-abdominal hypertension (dotted bars).  Asterisk: *P *< 0.05 vs positive end-expiratory pressure (PEEP) 5; Single Triangle: *P *< 0.05 PEEP 10 vs PEEP 15; Double Triangle: *P *< 0.05 PEEP 10 vs PEEP 20; Triple Triangle: *P *< 0.05 PEEP 15 vs PEEP 20. Data are presented as mean ± standard deviation. ZEEP = zero end-expiratory pressure.

At each level of PEEP, IAP was higher in the IAH group and increased with PEEP in both groups (Figure 2). End-inspiratory plateau pressure as well as end-inspiratory and end-expiratory Ptranspul increased with PEEP without differences between groups (Table 3). PEEP at 10 and 15 cmH_2_O decreased Estat, RS and Estat, L in patients with IAH, although not in those with normal IAP (Figures 3a, b). Over the range of PEEP studied, no changes in Estat, CW were observed in either group (Figures 3c). Exhaled volume from PEEP to ZEEP increased with PEEP, but it was not different between the two groups (Figure 3d).

**Table 3:** Effect of PEEP on gas exchange and respiratory mechanics

		PEEP 20	PEEP 15	PEEP 10	PEEP 5	*P *< 0.05 PEEP-level between groups:
**PaO_2_/FiO_2_**	**Normal IAP**	238.67 ± 87.84^**c**^	232.65 ± 85.17	191.33 ± 40.59	169.85 ± 44.09	NS
	**IAH**	218.72 ± 70.13^**c**^	208.67 ± 67.54^**e**^	181.25 ± 55.45	161.13 ± 47.59	
**PaCO_2_, mmHg**	**Normal IAP**	46.44 ± 8.20	46.40 ± 8.18	46.85 ± 8.12	46.88 ± 8.17	NS
	**IAH**	45.64 ± 8.44	47.00 ± 8.22	44.55 ± 7.43	45.27 ± 8.31	
**pH**	**Normal IAP**	7.34 ± 0.1	7.34 ± 0.11	7.34 ± 0.10	7.34 ± 0.11	NS
	**IAH**	7.37 ± 0.06	7.38 ± 0.06	7.39 ± 0.04	7.39 ± 0.06	
**VT (ml/kg IBW)**	**Normal IAP**	5.35 ± 0.43	5.36 ± 0.39	5.30 ± 0.43	5.31 ± 0.40	NS
	**IAH**	5.53 ± 0.30	5.58 ± 0.28	5.59 ± 0.32	5.59 ± 0.30	
**RR, breaths/min**	**Normal IAP**	18.30 ± 3.20	18.30 ± 3.20	18.30 ± 3.20	18.30 ± 3.20	NS
	**IAH**	16.70 ± 3.20	16.70 ± 3.20	16.90 ± 3.21	16.90 ± 3.21	
**PEEP, cmH_2_O**	**Normal IAP**	20.00 ± 0.00	15.00 ± 0.00	10.00 ± 0.00	5.00 ± 0.00	20,15,10,5
	**IAH**	20.00 ± 0.00	15.00 ± 0.00	10.00 ± 0.00	5.00 ± 0.00	
**Pplat, cmH_2_O**	**Normal IAP**	36.30 ± 6.40^**a, b, c**^	28.90 ± 3.21^**d, e**^	23.50 ± 2.92^**f**^	19.60 ± 3.66	NS
	**IAH**	35.80 ± 3,77^**a, b, c**^	29.20 ± 3.16^**d, e**^	24.50 ± 3.50^**f**^	22.10 ± 4.70	
**Pes insp, cmH_2_O**	**Normal IAP**	22.70 ± 5.83^**a, b, c**^	20.00 ± 3.94^**d, e**^	17.80 ± 3.71^**f**^	16.90 ± 4.36	NS
	**IAH**	24.69 ± 2.92^**a, b, c**^	21.15 ± 3.42^**e**^	19.25 ± 4.44^**f**^	18.85 ± 6.48	
**Pes exp, cmH_2_O**	**Normal IAP**	16.40 ± 4.50^**a, b, c**^	14.50 ± 3.63^**d, e**^	12.50 ± 3.31^**f**^	11.20 ± 3.52	NS
	**IAH**	16.91 ± 3.57^**a, b, c**^	14.35 ± 2.58^**d, e**^	12.55 ± 3.25^**f**^	11.65 ± 3.53	
**Ptranspul insp, cmH_2_O**	**Normal IAP**	16.60 ± 9.65^**a, b, c**^	11.90 ± 5.11^**d, e**^	8.70 ± 3.33^**f**^	5.70 ± 3.74	NS
	**IAH**	13.64 ± 3.75^**a, b, c**^	11.05 ± 3.88^**d, e**^	8.25 ± 4.97	6.25 ± 5.47	
**Ptranspul exp, cmH_2_O**	**Normal IAP**	6.60 ± 4.50^**a, b, c**^	3.50 ± 3.63^**d, e**^	0.50 ± 3.31^**f**^	-3.20 ± 3.52	NS
	**IAH**	6.09 ± 3.57^**a, b, c**^	3.65 ± 2.58^**d, e**^	0.45 ± 3.25^**f**^	-3.65 ± 3.53	

FiO_2 _= fraction of inspired oxygen; IAH = intra-abdominal hypertension; IAP = intra-abdominal pressure; IBW = ideal body weight; NS = not significant; PaCO_2 _= partial pressure of arterial carbon dioxide; PEEP = positive end-expiratory pressure; Pes exp = expiratory esophageal pressure; PaO_2 _= partial pressure of arterial oxygen; Pes insp = inspiratory esophageal pressure; Pplat = inspiratory plateau pressure; Ptranspulexp = expiratory transpulmonary pressure; Ptranspul insp = inspiratory transpulmonary pressure; RR = re spiratory rate; V_T _= tidal volume.

^**a**^*P *< 0.05 PEEP 20 vs. PEEP 15; ^**b**^*P *< 0.05 PEEP 20 vs. PEEP 10; ^**c**^*P *< 0.05 PEEP 20 vs. PEEP 5; ^**d**^*P *< 0.05 PEEP 15 vs. PEEP 10; ^**e**^*P *< 0.05 PEEP 15 vs. PEEP 5: ^**f**^*P *< 0.05 PEEP 10 vs. PEEP 5.

Data are presented as mean ± standard deviation

**Figure 4:**
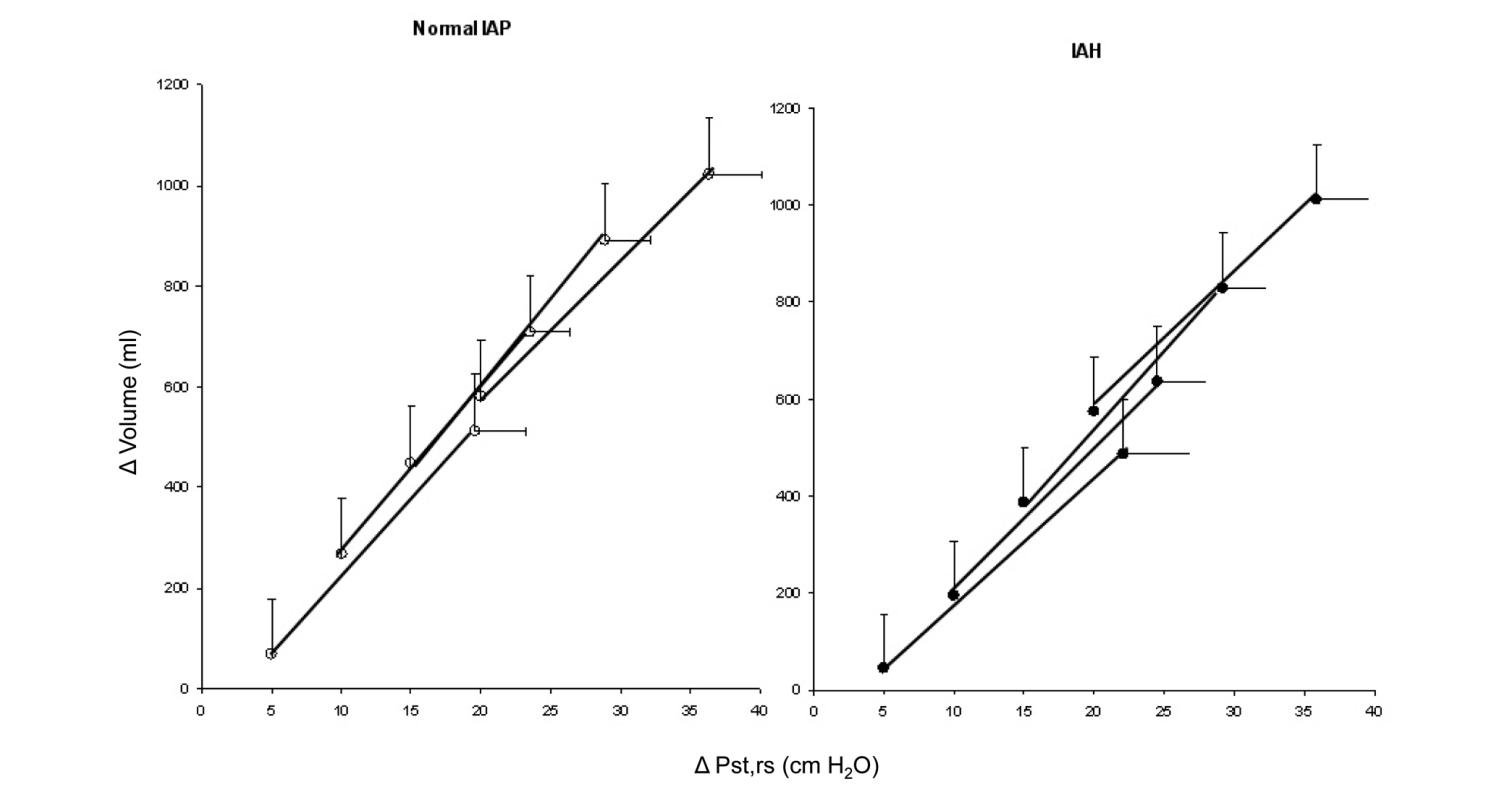
Pressure-volume (P-V) relationship in patients with normal intra-abdominal pressure (IAP; *left panel*) and intra-abdominal hypertension (IAH; *right panel*) as a function of PEEP.  The progressive shift to the left (i.e. at the same pressure the volume is higher at higher positive end-expiratory pressure (PEEP)) with increasing PEEP suggest recruitment. Data are presented as mean ± standard deviation.

The static tidal pressure-volume relationships at different PEEP levels in patients with and without IAH showed a progressive shift to the left indicating alveolar recruitment (Figure 4). However, alveolar recruitment from PEEP 5 cmH_2_O, computed at standardized pressure of 20 cmH_2_O was comparable at each level of PEEP in both groups (PEEP 10 cmH_2_O: 82 ± 113 vs. 51 ± 91 ml, PEEP 15 cmH_2_O: 88 ± 118 vs. 86 ± 115 ml, PEEP 20 cmH_2_O: 52 ± 123 vs. 131 ± 20 ml, respectively in normal IAP and IAH groups).

Higher PEEP improved oxygenation to a similar extent in both groups. For both groups, given levels of PEEP resulted in comparable levels of mean end-expiratory Ptranspul (Table 3). The PEEP-induced changes in end-expiratory Ptranspul were closely related to the changes in mean partial pressure of arterial oxygen (PaO_2_)/FiO_2 _ratio without significant differences for the two groups (r^2 ^= 0.88).

**Figure 5:**
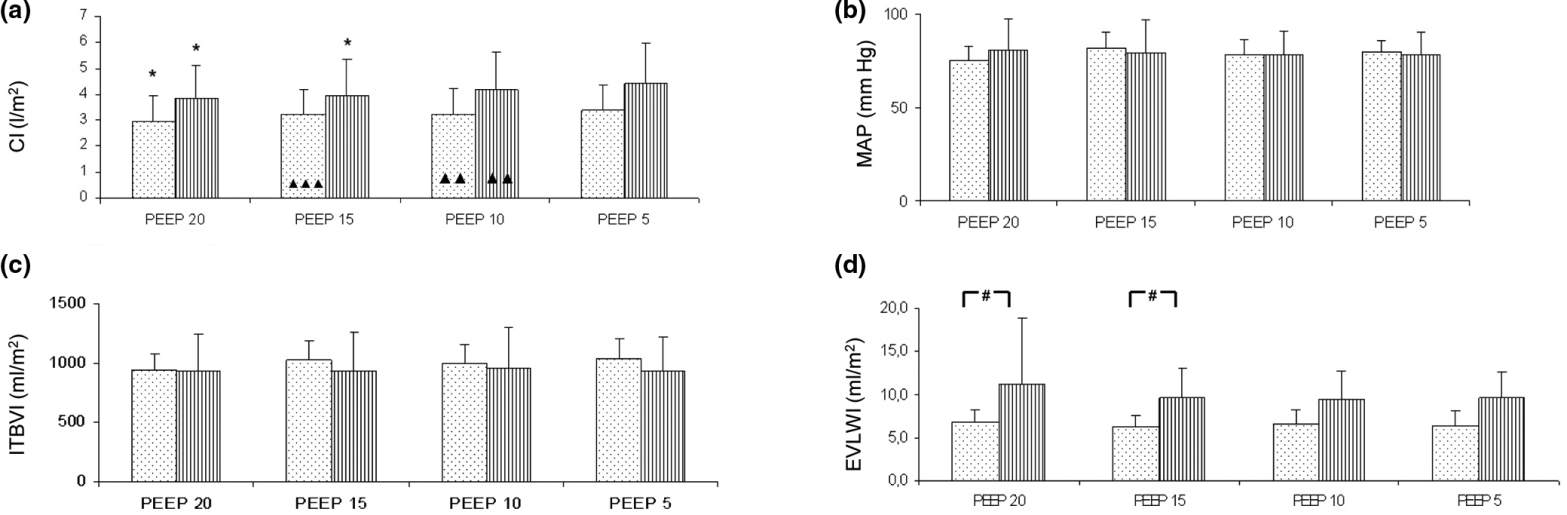
Effect of PEEP on (a) cardiac index (CI), (b) mean arterial pressure (MAP), (c) intrathoracic blood volume index (ITBI), and (d) extravascular lung water index in patients with normal normal intra-abdominal pressure (shaded bars) and intra-abdominal hypertension (dotted bars).  Asterisk: *P *< 0.05 vs positive end-expiratory pressure (PEEP) 5; Single Triangle: *P *< 0.05 PEEP 10 vs PEEP 15; Double Triangle: *P *< 0.05 PEEP 10 vs PEEP 20; Triple Triangle: *P *< 0.05 PEEP 15 vs PEEP 20; Hash key: *P *< 0.05 between groups. Data are presented as mean ± standard deviation.

Higher PEEP reduced CI in both IAP groups, while not affecting heart rate, MAP and intrathoracic blood volume (Figures 5a to 5c). Of note, EVLWI was higher at higher levels of PEEP in normal IAP compared with IAH group (PEEP 20: 11.2 ± 7.7 versus 6.8 ± 1.5 ml/m^2^, *P *< 0.05 between groups, Figure 5d).

In our study population 15 patients were classified as extrapulmonary ALI/ARDS while five as pulmonary ALI/ARDS. No significant differences were found in IAP, oxygenation or Estat, RS between groups at baseline or in response to PEEP.

## Discussion

In this prospective study, we found that ALI/ARDS patients with IAH, as compared with those with normal IAP, were characterized by: no differences in gas exchange, respiratory mechanics, partitioned into its lung and chest wall components, as well as hemodynamics and EVLWI/ITBVI, at comparable ventilator settings; and decreased Estat, RS and Estat, L, but no differences in alveolar recruitment and oxygenation or hemodynamics, when PEEP was increased at 10 and 15 cmH_2_O. However, at higher levels of PEEP, EVLWI was lower in ALI/ARDS patients with IAH as compared with those with normal IAP. Furthermore, we observed an increased ICU mortality and the prevalence of extrapulmonary ALI/ARDS in higher IAP group, although this was not the primary endpoint of the study.

Increased IAP markedly affects the function of different organs [1], particularly the mechanical properties of the respiratory system, lung and chest wall, and the respiratory function in different experimental settings [14] and in patients with ALI/ARDS [7], with a positive correlation between the Estat, CW and the IAP levels. Estat, CW was reported higher in surgical compared with medical ALI/ARDS patients [23], but IAP was not measured.

The present paper differs from the previous ones in the following issues: ALI/ARDS patients were prospectively stratified *a priori *according to their levels of IAP. We decided to use the threshold level of 12 mmHg to identify patients with IAH as compared with patients with normal IAP, as suggested by the Guidelines of the WSACS [2,3]; respiratory mechanics, partitioned into its lung and chest wall components, gas exchange, hemodynamics and EVLWI were simultaneously measured at comparable ventilator settings and at different levels of PEEP.

Several factors may explain the differences with data reported in previous studies. First, the range of IAP investigated in the study by Gattinoni and colleagues [7] was wider and the differences in mean IAP between patients with pulmonary and extrapulmonary ALI/ARDS were more pronounced (8.5 ± 2.9 vs 22.2 ± 6 cmH_2_O). Thus, it is possible that the effects on the chest wall mechanics may become apparent only at higher IAP levels. Second, in the present paper the patients were stratified for the level of IAP and not for the etiology. Third, respiratory mechanics was evaluated while patients were ventilated with protective tidal volumes, lower than that used in the previous studies.

Our findings also indicate that there was no effect of IAP on Pes expiration, as we previously found in healthy animals [14]. This finding may suggest that the transmission of the pressure at end expiration was not affected by IAH. Physiologic experimental studies [24,25] have shown that the deformation of the chest wall and the lung shape may compensate for significant changes in the pleural pressure and chest wall mechanical properties. In this line, in humans changes in chest wall mechanics are relatively small in healthy awake [26] and mechanically ventilated [27,28] subjects due to adaptability of total chest wall mechanical behavior at different IAP. Our data suggest that such compensatory mechanisms may be effective in ALI/ARDS mechanically ventilated patients at least for IAP pressure below 20 mmHg. Gas exchange was not different at PEEP 5 cmH_2_O between groups. It has been reported that decompression of the abdomen in surgical ALI/ARDS patients was associated with an improved oxygenation [23]. We did not find differences in ITBVI, EVLWI and hemodynamics between ALI/ARDS patients with and without IAH. Our patients were characterized by relatively low EVLWI in line with previous findings [29]. Groeneveld and Verheij [29], for example, reported relatively low EVLWI in ALI/ARDS with a tendency toward lower EVLWI in patients with extrapulmonary sepsis (7.8 ml/kg) compared with pneumonia (9 ml/kg). It has recently been pointed out by Gattinoni and Caironi [30] that a substantial proportion of ALI/ARDS patients present only a modest degree of lung edema and collapse. EVLWI in our study was relatively low, which may be due to the fact that fluid therapy was titrated aiming at low normal values of ITBVI, avoiding fluid excess with consecutive interstitial edema. Surprisingly, the level of EVLWI was even lower in this group at higher levels of PEEP.

EVLWI measurements deserve careful consideration. First, the single-indicator thermal dilution method, even if shown to correlate quite well with the reference gravimetric method in animal models (reviewed by [31]), has its inherent limitations. Second, the effect of PEEP on EVLWI measurements is still controversial with studies showing a decrease, increase or no change with PEEP (reviewed in [31]). We speculate that the effect of PEEP on EVLWI was rather negligible in our patients for the following reasons. First, using low tidal volume ventilation, Ptranspul at end inspiration as the clinical surrogate of lung stress during mechanical ventilation [8] was low even at the highest level of PEEP studied for both groups. Second, the duration of our study was rather short. Third, EVLWI measured during the decremental trial at PEEP 10 cmH_2_O (i.e. after ventilation with high PEEP) was comparable with baseline measurements taken at a PEEP of 9.1 ± 1.7 and 9.6 ± 1.8 cmH_2_O, respectively. The increase in EVLWI observed at high levels of PEEP may be due to a redistribution of blood flow and hence may falsely signal an increase in edema [32].

We would have expected that IAH was associated with an increase in ITBVI and EVLWI. In ALI [14] and septic [33] animal models, IAP increases in ITBVI and edema are likely to be due to higher microvascular permeability [34-36].

We observed that with increasing PEEP, IAP increased from 7.7 ± 3.7 5 mmHg at PEEP 5 cmH_2_O to 9.8 ± 4 mmHg at PEEP 20 cmH_2_O in ALI/ARDS patients without IAH and from 12.6 ± 4.2 to 15.5 ± 3.4 mmHg with IAH, respectively. This relatively small PEEP-induced increase in IAP is in keeping with previous findings by de Keulenaer and colleagues [37]. Gattinoni and colleagues [7] reported a reduction in the Estat, RS, Estat, L and Estat, CW associated with higher recruitment in patients with higher IAP. On the contrary, we found that PEEP at 10 and 15 cmH_2_O decreased the Estat, RS and Estat, L, but not Estat, CW in ALI/ARDS with IAH. No significant effects of PEEP on respiratory mechanics were observed in ALI/ARDS with normal IAP. This observation may be explained by an increase in lung volume by PEEP or recruitment of previously collapsed alveoli. The limited amount of recruitment in both groups may be explained by the low EVLWI as indicated above, suggesting minor alveolar edema and atelectasis. Our data are also in line with those reported by Thille and colleagues [38] showing no differences in respiratory mechanics between pulmonary and extrapulmonary ALI/ARDS, with an amount of recruitment also being relatively low.

It has been hypothesized that in ALI/ARDS patients with IAH the hemodynamic response to PEEP is different as compared with ALI/ARDS with normal IAP. Some authors [7] hypothesized that since in ALI/ARDS increased PEEP improved Estat, CW in patients with IAH, thus reducing pleural pressures, this was associated with better hemodynamics as compared with those patients with lower IAP and less recruitment. On the other hand, it could be expected that patients with IAH may show a decreased cardiac output and impaired hemodynamics when PEEP is applied, due to a reduction in Estat, CW. We did not observe any significant effect of IAH on hemodynamics and especially on ITBVI. This can clearly be explained by the fact that we did not observe any effect of IAH on the Estat, CW and thus in the changes in pleural pressure and thus intrathoracic pressure.

It has also been suggested that the measurement of ITBVI could be a more accurate measurement of preload as compared with conventional intravascular pulmonary pressures in presence of IAH [6]. This is due to the fact that the real transcardiac pressures may be affected by changes in Estat, CW and thus pleural pressures in presence of IAH. Again as we did not observe any relevant effect of IAH on Estat, CW and pleural pressures estimated by Pes, we believe that IAH does not markedly influence transcardiac pressures.

Some limitations of the present study must be discussed to better interpret the results. First, the sample size was limited, so this study is just hypothesis generating rather than confirmative. Second, the range of IAP of the patients included in the study was rather limited, thus we cannot exclude that in presence of IAP higher than 20 mmHg more significant effects could have been observed. Third, the definition of normal IAP for the supine patient is somewhat questionable [37]. De Keulenaer and colleagues [37] state that normal IAP in the general population ranges from 5 to 7 mmHg, also suggesting that normal values of IAP in the obese patients should be considered as between 7 and 14 mmHg. As the 'normal IAP' patients in our study had a BMI of 29 ± 4 kg/m^-2^, an IAP of 8 ± 3 mmHg may in fact be regarded as normal. However, we used the classical definition of IAH as proposed by WSACS [2,3]. Fourth, PEEP levels were not randomized, but rather studied in a decremental fashion. This approach, combined with recruitment maneuvers before each PEEP setting in order to standardize lung volume history, is part of the open lung ventilation strategy in our unit [11]. Fifth, we did not separate between pulmonary and extrapulmonary primary and secondary ALI/ARDS because our intent was to investigate the effects of IAH, independent of the cause of the disease on respiratory function effects induced by PEEP. Sixth, we did not use paralyzing agents throughout the study, but respiratory muscle activity could be excluded by the analysis of the Pes curves. Finally, the observations were limited in time.

## Conclusions

We found that in patients with ALI/ARDS, IAH did not markedly affect respiratory system mechanics, gas exchange, hemodynamics and EVLWI. PEEP did not affect respiratory mechanics, alveolar recruitment, gas exchange and hemodynamics in ALI/ARDS patients with normal IAP or IAH. However, PEEP significantly increased EVLWI in patients with normal IAP but not in patients with IAH. IAH, within the limits of IAP measured in the present study, does not affect interpretation of respiratory mechanics and hemodynamics. Pending further studies, these data suggest that partitioning of respiratory mechanics should not be routinely performed in mechanically ventilated ALI/ARDS patients and that PEEP should not be selected on the basis of IAP levels.

## Key messages

• Moderate IAH does not affect respiratory system mechanics, gas exchange, hemodynamics and EVLWI.

• PEEP does not differently affect respiratory mechanics, alveolar recruitment, gas exchange and hemodynamics in ALI/ARDS patients with normal IAP or IAH.

• IAH, within the limits of IAP measured in the present study, does not affect interpretation of respiratory mechanics and hemodynamics.

## Abbreviations

ALI: acute lung injury; ARDS: adult respiratory distress syndrome; CI: cardiac index; Estat, CW: static chest wall elastance; Estat, L: static lung elastance; Estat, RS: static respiratory system elastance; EVLWI: extravascular lung water index; FiO_2_: fraction of inspired oxygen; IAH: intra-abdominal hypertension; IAP: intra-abdominal pressure; ITBVI: intrathoracic blood volume index; MAP: mean arterial pressure; PaO_2_: partial pressure of arterial oxygen; PEEP: positive end-expiratory pressure; Pes: esophageal pressure; Ptrach: tracheal pressure; Ptranspul: transpulmonary pressure; SAPS: Simplified Acute Physiology Score; WSACS: World Society of Abdominal Compartment Syndrome; ZEEP: zero end-expiratory pressure

## Competing interests

The authors declare that they have no competing interests.

## Authors' contributions

JK, PP and TL participated in the study design. JK, CT, MA and TL performed the study. JK, PP and TL processed the data and performed the statistical analysis. TL and PP wrote the manuscript. All authors read and approved the final manuscript.
